# Efficacy of Anti-PD-1/PD-L1 Monotherapy or Combinational Therapy in Patients Aged 75 Years or Older: A Study-Level Meta-Analysis

**DOI:** 10.3389/fonc.2021.538174

**Published:** 2021-03-19

**Authors:** Run-Cong Nie, Guo-Ming Chen, Yun Wang, Jie Zhou, Jin-Ling Duan, Zhi-Wei Zhou, Shu-Qiang Yuan

**Affiliations:** ^1^ Department of Gastric Surgery, Sun Yat-sen University Cancer Center, State Key Laboratory of Oncology in South China, Collaborative Innovation Center for Cancer Medicine, Guangzhou, China; ^2^ Department of Hematologic Oncology, Sun Yat-sen University Cancer Center, State Key Laboratory of Oncology in South China, Collaborative Innovation Center for Cancer Medicine, Guangzhou, China; ^3^ Department of Experimental Research, Cancer Institute, Sun Yat-sen University Cancer Center, State Key Laboratory of Oncology in South China, Collaborative Innovation Center for Cancer Medicine, Guangzhou, China

**Keywords:** programmed cell death 1 (PD-1), PD-L1, age, efficacy, overall survival

## Abstract

Recent trials have shown a promising anti-tumor activity for advanced cancer patients treated with PD-1/PD-L1 inhibitors; however, little is known on the use of PD-1/PD-L1 inhibitors in adults over 75 years of age. Here, we performed a study-level meta-analysis to compare the efficacy of anti-PD-1/PD-L1 agents between elderly (≥ 75 years) and non-elderly (< 75 years) patients. In the present study, we systematically reviewed phase 2/3 trials of PD-1/PD-L1 inhibitors of advanced solid tumors that reported treatment effect (hazard ratio [HR]) in patients based on age (≥ 75 years vs. < 75 years) and set anti-PD-1/PD-L1 monotherapy or combinational therapy as experimental arm. The HRs of OS and progression-free survival (PFS) are based on random-effect models. Overall, a total of eight qualifying trials comprising 5,393 subjects were included for meta-analysis, and 472 patients (8.8%) were aged 75 years or older. The overall estimated HR for OS was 0.70 (0.62–0.79) in patients < 75 years vs. 0.94 (0.67–1.30) in patients ≥ 75 years. Anti-PD-1/PD-L1 agents improved OS of melanoma patients in both elderly (HR 0.25 [0.10-0.60]) and non-elderly (HR 0.49 [0.33–0.71]) group. The OS difference in the efficacy of PD-1/PD-L1 inhibitors between elderly and non-elderly patients was significant (P = 0.043 for interaction). The overall estimated HR for PFS was 0.77 (0.60–1.00) in patients < 75 years vs. 0.97 (0.60–1.58) in patients ≥ 75 years. Therefore, with the exception of melanoma, elderly patients (≥ 75 years) could not benefit from the anti-PD-1/PD-L1 agents in survival, and toxicity profile of anti-PD-1/PD-L1 drugs should be explored in this population.

## Introduction

Recently, the advent of immune checkpoint inhibitors (ICIs) has tremendously revolutionized the landscape of cancer treatment. Monoclonal antibodies targeting the programmed cell death 1 (PD-1) or its ligand, programmed death ligand 1 (PD-L1), have been demonstrated to induce remarkable survival outcomes in a wide range of advanced malignancies (1–10). So far, the FDA has approved anti-PD-1/PD-L1 agents and their combinations for treatment of more than 13 cancer types and mismatch repair deficiency or microsatellite instable-high solid tumors (11). However, although cancer predominantly affects older adults (12), there is a lack of consensus on the efficacy of anti-PD-1/PD-L1 immunotherapy in this geriatric population (13).

It had been reported that aging is accompanied by decreased or dysregulation of immunity (14–16), which can theoretically blunt the efficacy of the PD-1/PD-L1 inhibitors. There have no prospective clinical trials focused specifically on the use of PD-1/PD-L1 inhibitors in elderly patients, which was mainly due to concerns about the safety profile. Elias et al. (17) also reported that PD-1/PD-L1 inhibitors had similar therapeutic effect in younger and older patients, with an age cutoff of 65 years. With the aging of society, average human lifespan has dramatically increased across the globe (12), with life expectancy higher than 75 years, and the therapeutic effect of PD-1/PD-L1 inhibitors in patients aged 75 years or older was still unknown. Therefore, we conducted a study-level meta-analysis to compare the efficacy of anti-PD-1/PD-L1 agents between elderly (≥ 75 years) and non-elderly (< 75 years) patients.

## Methods

### Search Strategy and Study Selection

This study was conducted in compliance with Cochrane Handbook for Systematic Reviews of Interventions recommendations and was reported based on Preferred Reporting Items for Systematic Reviews and Meta-Analyses (PRISMA) statement guidelines (18).

We conducted a comprehensive systematic search of Medline (PubMed), Embase, ClinicalTrials.gov and Cochrane Library databases from inception to November 2019 using the following key words: “pembrolizumab”, “nivolumab”, “avelumab”, “atezolizumab”, “durvalumab”, and “immune checkpoint inhibitor”, limiting to phase 2 trials and phase 3 trials. The 2019 ASCO meeting and 2019 ESMO congress were also searched for the additional studies. The terms of the search strategy were shown in **Doc S1** (**Supplementary Materials**). The clinical trials should meet the following criteria: (1) phase 2 and phase 3 randomized controlled trials (RCTs) of advanced solid tumors; (2) studies that assigned participants to PD-1/PD-L1 inhibitors or non-PD-1/PD-L1 inhibitors, and set anti-PD-1/PD-L1 monotherapy or combinational therapy as experimental arms; (3) subgroup comparisons of overall survival (OS) using a hazard ratio (HR) based on the age (≥ 75 years versus < 75 years). Two independent authors (RCN and YFL) screened the titles and abstracts of the reports to identify the potential articles, and then assessed the eligibility of the full texts of these relevant articles. The references of the relevant trials were also reviewed through hand-searching strategy.

### Data Extraction

Two reviewers (RCN and YFL) extracted the following data: first author’s name, study number, accrual period, phase of study, included population, line of therapy, treatment strategy, number of patients by age, median follow-up duration, HR for OS and for progression-free survival (PFS) based on the age. The discrepancies in the literature search and data extraction were resolved by consensus.

We also used the MSKCC immunotherapy cohort (19) to further explore the tumor mutation burden (TMB) and survival outcomes according to the age through cBioPortal website (https://www.cbioportal.org/). We extracted the data of anti-PD1 monotherapy or combination therapy of the specified cancer types that were included in the meta-analysis.

### Data Synthesis and Analysis

In our study, the primary outcome was to evaluate OS between elderly and non-elderly patients treated with PD-1/PD-L1 inhibitors. The secondary outcome was to compare the PFS between elderly and non-elderly patients. The measures of OS and PFS were quantified by the HR with the corresponding 95% confidence intervals (CI). The heterogeneity among trials was examined using Cochran Q statistic, and quantified by I^2^ index. The heterogeneity was considered significant for P < 0.1 and I^2^ < 50%. All the pooled HR of this meta-analysis was calculated through random effects model because of the potential heterogeneity among the included trials. For studies that reported the HR estimates for < 65 and 65–75 years separately, we combined the estimate (< 75 years) using a random effects model. An interaction test was used to evaluate the heterogeneity of efficacy between subgroups, which was expressed as P for interaction to quantify the potential publication bias. Regarding the MSKCC immunotherapy cohort, TMB and OS based on the age were compared by t-test and log-rank test, respectively. All the statistical analyses were performed using Stata version 13.0 (StataCorp, College Station, TX) and R version 3.6.2 (https://www.r-project.org/). Statistical significance was considered as P < 0.05.

## Results

### Search Results and Patient Characteristics

**Figure 1:**
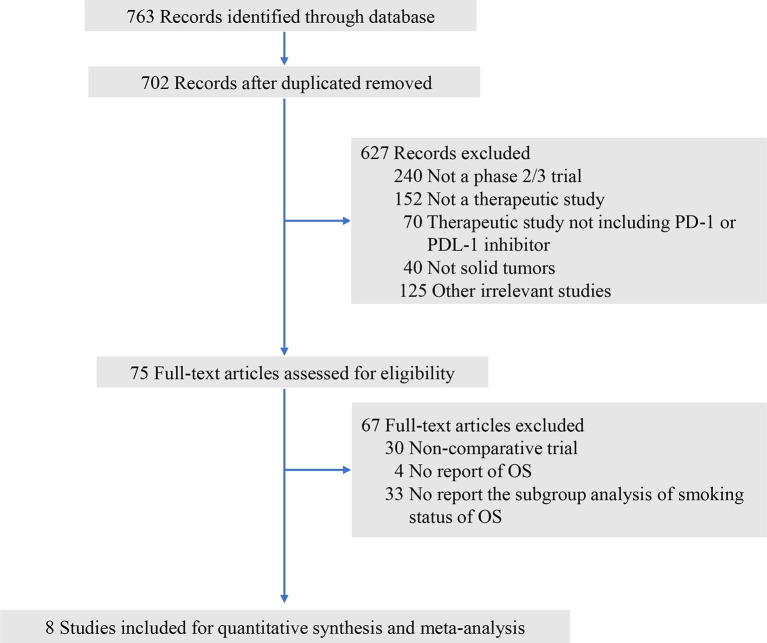
Study flow diagram. OS, overall survival; PD-1, programmed death 1; PD-L1, programmed death-ligand 1.

Initially, a total of 763 relevant publications matched our basic search strategy. After screening the titles and abstracts of these publications, 75 studies were reviewed the full-text for eligibility. Sixty-seven of those 75 articles were excluded since they did not report the OS subgroup by age with the cutoff 75 years (**Figure 1**). Finally, a total of eight phase 3 trials (1, 8, 20–25) were included for meta-analysis, among which four investigated nivolumab, two investigated the combination of nivolumab plus ipilimumab, one investigated pembrolizumab, and one investigated avelumab. The underlying malignancies comprised were non-small-cell lung cancer (four trials), renal-cell carcinoma (two trials), melanoma (one trial), and head and neck cancer (one trial). There were six trials of anti-PD-1/PD-L1 monotherapy and two trials of anti-PD-1/PD-L1 plus anti-CTLA therapy as the experimental arms. A total of 5,393 patients (intervention: 2,698; control: 2,695) were included, and 472 patients (8.8%) were aged 75 years or older. The baseline characteristics of each trial are presented in **Table 1**. A funnel plot was performed to assess the publication bias, which showed a symmetric distribution of studies on either side of the funnel. The Begg and Egger test also indicated no obvious publication bias (P = 0.071; **Figure S1**).

**Table 1:** Characteristics of the included trials.

Studies	Study number	Trial period	Population	Line of therapy	Experimental arm	Control arm	Patients number	N (%) < 75 years	N (%) ≥ 75 years	Follow-up (months)
Borghaei et al. (1)	CheckMate 057	2013–2013	Nonsquamous NSCLC	1–3	Nivolumab	Docetaxel	582	539 (92.6)	43 (7.4)	13.2
Brahmer et al. (20)	CheckMate 017	2012–2013	Squamous NSCLC	2	Nivolumab	Docetaxel	272	243 (89.3)	29 (10.7)	11.0
Motzer et al. (21)	CheckMate 025	2012–2014	Renal-Cell Carcinoma	2-3	Nivolumab	Everolimus	821	747 (91.0)	74 (9.0)	14.0
Robert et al. (22)	CheckMate 066	2013–2014	Melanoma	2	Nivolumab	Dacarbazine	481	414 (86.1)	67 (13.9)	16.7
Barlesi et al. (23)	JAVELIN Lung 200	2015–2017	NSCLC	1–3	Avelumab	Docetaxel	529	479 (90.5)	50 (9.5)	18.3
Motzer et al. (24)	CheckMate 214	2014–2016	Renal-Cell Carcinoma	1	Nivolumab +ipilimumab	Sunitinib	847	782 (92.3)	65 (7.7)	25.2
Cohen et al. (8)	KEYNOTE-040	2014–2016	Head and neck cancer	> 2	Pembrolizumab	ICC	495	464 (93.7)	31 (6.3)	7.5
Hellmann et al. (25)	CheckMate 227	2015–2016	NSCLC	1	Nivolumab +ipilimumab	Chemotherapy	1,166	1,053 (90.3)	113 (9.7)	29.3

NSCLC, non-small-cell lung cancer; ICC, investigator’s choice-chemotherapy.

### Primary Outcome: Overall Survival

**Figure 2:**
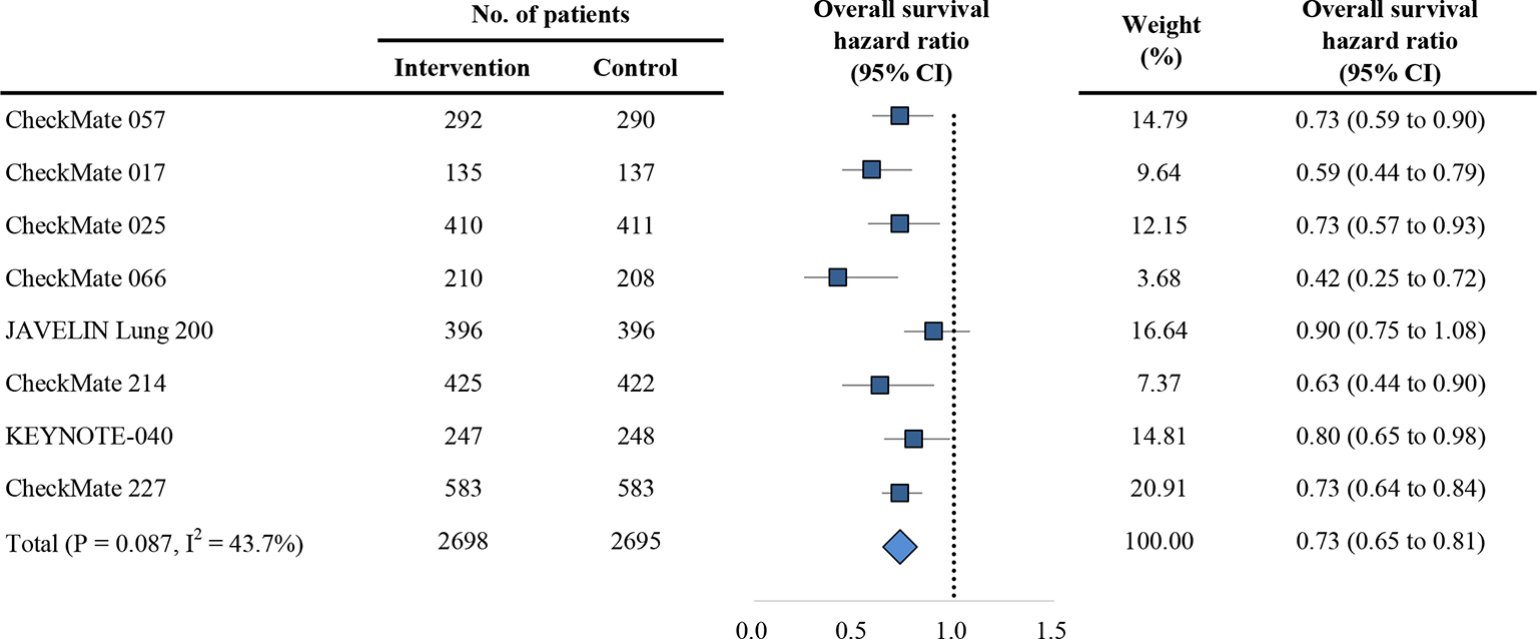
Forest plot of hazard ratio for overall survival. CI, confidence interval; PD-1, programmed death 1; PD-L1, programmed death-ligand 1.

**Figure 3:**
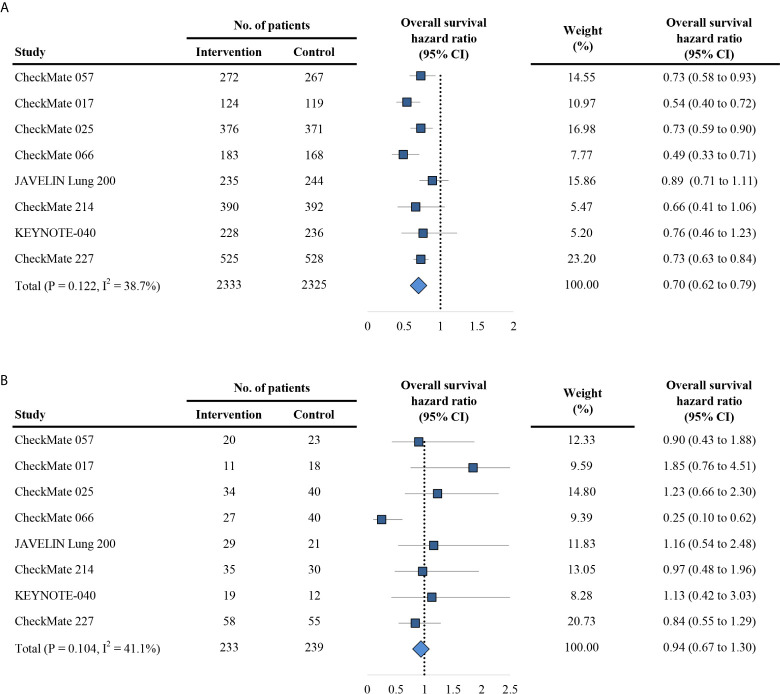
Forest plot of hazard ratio for overall survival in patients < 75 years **(A)** and ≥ 75 years **(B)**. CI, confidence interval; PD-1, programmed death 1; PD-L1, programmed death-ligand 1.

The primary outcome is OS in trials comparing PD-1/PD-L1 inhibitors with control agents. The HR of each trial and the pooled result based on the random effects model are shown in **Figure 2**. Overall, the estimated HR is 0.73 (95% CI: 0.65 to 0.81) (P < 0.001), suggesting that PD-1/PD-L1 inhibitors reduced the risk of death by 27% compared with control treatments. Patients were then dichotomized into elderly and non-elderly groups with the cutoff of 75 years. For non-elderly patients, the estimated HR for OS showed significant difference between PD-1/PD-L1 inhibitors and control agents (HR, 0.70; 95% CI: 0.62 to 0.79; P < 0.001; **Figure 3A**). For this subgroup, no significant heterogeneity was observed among individual trials (I^2^ = 38.7%, chi-squared P = 0.122). For elderly patients, no significant heterogeneity was observed (I^2^ = 41.1%, chi-squared P = 0.104), and all studies reported no OS benefit for PD-1/PD-L1 inhibitors except for the CheckMate 066, which explored the efficacy of nivolumab in melanoma (22); the overall estimated HR was 0.94 (95% CI: 0.67 to 1.30; P = 0.696; **Figure 3B**). Furthermore, we observed a significant heterogeneity of efficacy between elderly and non-elderly patients concerning the pooled HRs for OS (P = 0.043 for interaction), which indicated that the effects of the anti-PD-1/PD-L1 therapy on OS varied for the elderly and non-elderly adults (**Table 2**). Subgroup stratified by anti-PD-1/PD-L1 monotherapy and anti-PD-1/PD-L1 combinational therapy showed the similar results (Table S1). Moreover, we found that the estimated HR for OS was 0.68 (95% CI: 0.59 to 0.77; P < 0.001; **Figure S2**) in patients aged 65-75 years.

**Table 2:** Summary of HR for PFS and OS by age.

Age	OS		PFS	
	HR (95% CI)	P for interaction*	HR (95% CI)	P for interaction*
<75 years	0.70 (0.62 to 0.79)	0.043	0.77 (0.60 to 1.00)	0.433
≥75 years	0.94 (0.67 to 1.30)		0.97 (0.60 to 1.58)	

HR, hazard ratio; PFS, progression-free survival; OS, overall survival; CI, confidence interval.

*P for interaction was expressed as the heterogeneity of efficacy between elderly and non-elderly patients.

### Secondary Outcome: Progression-Free Survival

**Figure 4:**
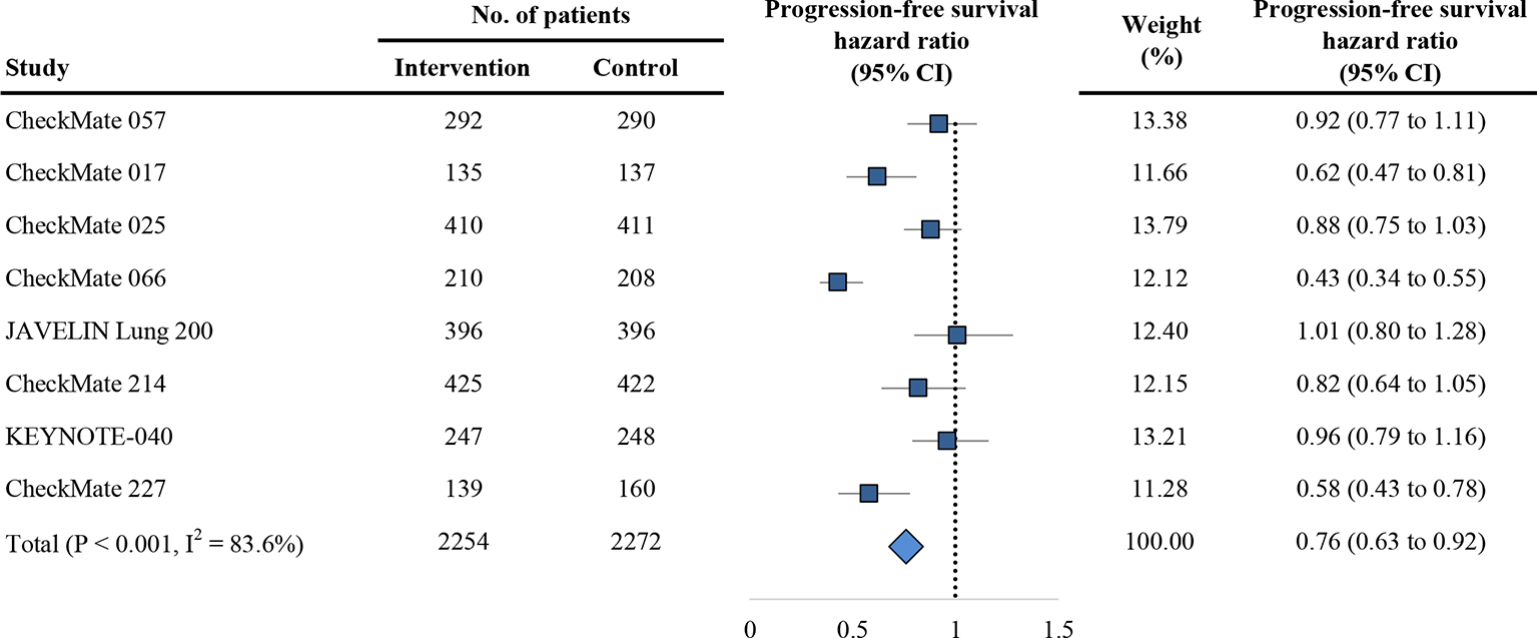
Forest plot of hazard ratio for progression-free survival. CI, confidence interval; PD-1, programmed death 1; PD-L1, programmed death-ligand 1.

**Figure 5:**
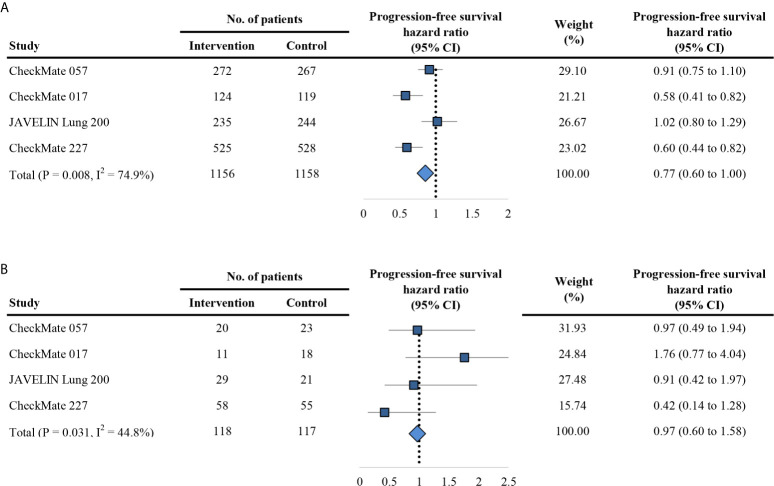
Forest plot of hazard ratio for progression-free survival in patients < 75 years **(A)** and ≥ 75 years **(B)**. CI, confidence interval; PD-1, programmed death 1; PD-L1, programmed death-ligand 1.

The secondary outcome is PFS in trials comparing PD-1/PD-L1 inhibitors with control agents. Of the eight included trials, eight reported HR for overall PFS, and four reported HR for PFS based on the age group. Overall, the estimated HR for PFS is 0.76 (95% CI: 0.63 to 0.92; P = 0.005; **Figure 4**), indicating of 24% reduction of the risk of progression treated with anti-PD-1/PD-L1 agents. Using the random effects model, the measures of PFS of the non-elderly patients favored anti-PD-1/PD-L1 therapy for a HR of 0.77 (95% CI: 0.60 to 1.00; P = 0.054; **Figure 5A**). There was significant heterogeneity among individual trials (I^2^ = 74.9%, chi-squared P = 0.008). However, for elderly patients, the PFS was not different between PD-1/PD-L1 inhibitors and controls (HR, 0.97; 95% CI: 0.60 to 1.58; P = 0.898; **Figure 5B**). No difference between elderly and non-elderly subsets was observed regarding the estimated HR for PFS (P = 0.433 for interaction) (**Table 2**).

### Tumor Mutation Burden

**Figure 6:**
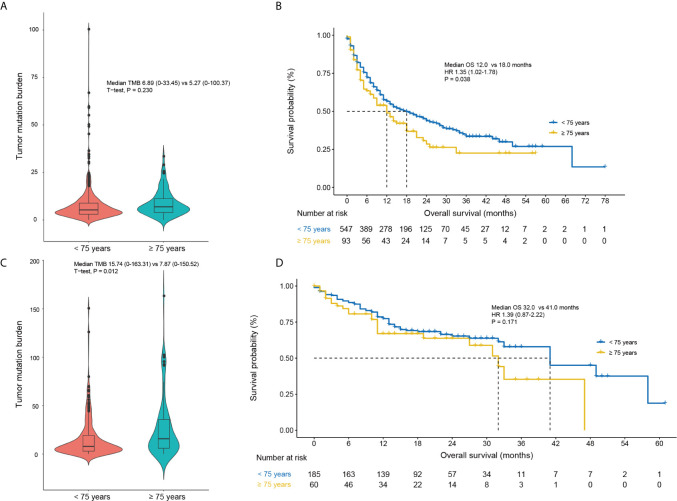
Comparisons of the tumor mutation burden and OS based on the age (< 75 years vs. ≥ 75 years) in non-melanoma **(A, B)** and melanoma patients **(C, D)** of the MSKCC immunotherapy cohort (19). CI, confidence interval; HR, hazard ratio.

Finally, we extracted the patients with non-small-cell lung cancer, renal-cell carcinoma, head and neck cancer and melanoma to explore the TMB differences between elderly and non-elderly patients in the MSKCC immunotherapy cohort (19). A total of 845 patients treated with PD-1/PD-L1 inhibitors were included. For non-melanoma cohort, the TMB between elderly and non-elderly patients was not significant (median TMB: 6.89 [0–33.45] vs 5.27 [0–100.37]; P = 0.230; **Figure 6A**), and elderly patients were associated with shorted OS (HR, 1.35; 95% CI: 1.02 to 1.78; P = 0.038; **Figure 6B**). Interesting, for melanoma cohort, elderly patients had higher TMB than non-elderly patients (median TMB: 15.74 [0–33.45] vs 7.87 [0–100.37]; P = 0.012; **Figure 6C**), and comparable OS with non-elderly patients (HR, 1.39; 95% CI: 0.87 to 2.22; P = 0.171; **Figure 6D**).

## Discussion

The aging phenomenon is one of the most significant global challenges today, which would accompany with age-related disease, such as increasing incidence of cancer. Aging is associated with the immune dysfunction that may affect the efficacy of PD-1/PD-L1 inhibitors (14, 15, 26, 27). Consequently, we performed this first meta-analysis of eight RCTs that comprised 5393 patients to explore the efficacy of PD-1/PD-L1 inhibitors in patients ≥ 75 years with metastatic cancer compared to those < 75 years. Overall, our finding suggested that the impact of PD-1/PD-L1 inhibitors significantly improved the OS (HR 0.70 [0.62–0.79] vs. 0.94 [0.67–1.30]) and PFS (HR 0.77 [0.60–1.00] vs. 0.97 [0.60–1.58]) in the non-elderly patients rather than elderly patients. Parallel results were confirmed in both monotherapy and combinational therapy. Melanoma patients aged 75 years or older can still benefit from PD-1/PD-L1 inhibitors.

Only two previous literatures attempt to review the topic of geriatric population in PD-1/PD-L1 inhibitors (17, 28). Nishijima et al. (28) compared the efficacy of ICIs between younger and older patients. However, only four of their included trials (eight trials) were anti-PD-1 trials. They concluded that a benefit in OS with ICIs was observed in both younger (HR, 0.75; 95% CI, 0.68–0.82) and older (HR, 0.73; 95% CI, 0.62–0.87) patients, but it should be noted that the age cutoff of this study was non-uniform (65–70 years). Elias et al. (17) also reported that anti-PD-1/PD-L1 agents had similar therapeutic effect in younger and older patients. Nonetheless, this analysis dichotomized patients into younger and older with an age cutoff of 65 years. Initially, we also found that patients aged 65–75 years could still benefit from PD-1/PD-L1 inhibitors. With the aging of society, adults over 75 years of age contribute more than 25% of the new cancer cases annually (12). Therefore, it is critical to clarify the efficacy of PD-1/PD-L1 inhibitors specific to patients aged 75 years or older. In contrary to the previous meta-analysis, we observed the better survival outcomes only in patients < 75 years, but not in patients ≥ 75 years. It had been reported that aging is associated with immune dysregulation, such as the decreased TCR diversity in CD4+ T cells (14) and CD8+ T cells (15), but not the T-cell immunosenescence (29). In addition, aging-associated adipocyte accumulation in the bone marrow also contributes to reduced hematopoiesis with age (30), and hematopoiesis becomes skewed toward myeloid and away from lymphoid lineages with age (16). Moreover, aging is associated with increased M2 polarization (26). Thus, the hypothesis of immunosenescence, the age-related decline in host immunity, may explain the invalid efficacy of PD-1/PD-L1 inhibitors in patients ≥ 75 years.

Among the eight included trials, the CheckMate 066 (22) study reported the OS outcomes of melanoma for the age groups of 75 years. Notably, we found that a significant improvement in OS was still observed in melanoma patients aged 75 years or older (HR, 0.25; 95% CI, 0.10–0.62; **Figure 3B**). Betof et al. (31) reported the survival outcomes of 254 patients with metastatic melanoma treated with anti-PD-1/PD-L1 agents, and found that the OS and PFS of patients ≥ 75 years were comparable to those with age < 75 years. Additionally, the safety profiles were also similar. In the present study, we also used the MSKCC immunotherapy cohort (19) to further explore the underlying mechanism of age based efficacy difference in term of TMB (**Figure 6**). Interestingly, an increased TMB was observed in the elderly patient with melanoma; this increased TMB may restore the age-related immune dysfunction of melanoma, thus leading to the comparable immunity between patients younger and older than 75 years.

Our study has several potential limitations. First, we observed heterogeneity among the included trials, which was mainly due to the multiple cancer types among the included trials. Thus, we minimized its influence through the random effects model for quantitative synthesis. However, the conclusion of this study still cannot be expanded to all solid tumor types. Second, although our work contributes the best level of evidence showing an age-based (≥ 75 years vs. < 75 years) efficacy difference for anti-PD-1/PD-L1 agents, this is a meta-analysis at study-level in essence. A meta-analysis at individual level should be performed to further validate the impact of age on the efficacy of anti-PD-1/PD-L1 agents. Third, the toxicities difference between elderly and non-elderly patient could not be analyzed because of lack of report. The toxicity profile might influence the therapeutic choice between anti-PD-1/PD-L1 agents or standard chemotherapy in elderly patients. Finally, elderly patients enrolled in clinical trials were a selected population with good performance status at academic hospitals. These selected elderly patients do not represent the real medical conditions of the elderly adults. Therefore, whether our findings applicable to patients treated in the community remains further validation.

In conclusion, our study suggested that the use of anti-PD-1/PD-L1 significantly improved the survival of patients aged < 75 years, but not those aged ≥ 75 years. This is mainly due to a potential interaction of immunosenescence and efficacy of anti-PD-1/PD-L1 drugs. An improved OS was still observed in melanoma patients aged ≥ 75 years, owing to the increased TMB in the elderly melanoma patients. Overall, our study indicated that, with the exception of melanoma, elderly patients (≥ 75 years) could not benefit from the anti-PD-1/PD-L1 agents in survival, and toxicity profile of anti-PD-1/PD-L1 drugs should be explored in this population.

## Data Availability Statement

The datasets generated for this study are available on request to the corresponding authors.

## Author Contributions

All authors contributed to the study concept and design. R-CN and G-MC extracted the data and performed the quality assessment. R-CN, YW and JZ participated in analysis of the data. R-CN, YW, J-LD and JZ contributed to drafting of the manuscript. S-QY and Z-WZ revised the manuscript. All authors contributed to the article and approved the submitted version.

## Conflict of Interest

The authors declare that the research was conducted in the absence of any commercial or financial relationships that could be construed as a potential conflict of interest.
