# Supplementary material for: Efficacy of Anti-PD-1/PD-L1 Monotherapy or Combinational Therapy in Patients Aged 75 Years or Older: A Study-Level Meta-Analysis
Source: Front Oncol. 2021 Mar 19;11:538174. doi: 10.3389/fonc.2021.538174 (PMC8018595; doi:10.3389/fonc.2021.538174)
Supplement: Supplementary file 1 [file DataSheet_1.docx]

**Doc S1: PubMed search terms**

(“nivolumab”[Supplementary Concept] OR “Nivolumab”[tiab] OR “Opdivo”[tiab] OR “MDX-1106”[tiab] OR “ONO-4538”[tiab] OR “BMS-936558”[tiab] OR “NIVO”[tiab] OR “pembrolizumab”[Supplementary Concept] OR “pembrolizumab”[tiab] OR “lambrolizumab”[tiab] OR “keytruda”[tiab] OR “MK-3475”[tiab] OR “SCH 900475”[tiab] OR “avelumab”[Supplementary Concept] OR “Avelumab”[tiab] OR “MSB0010718C”[tiab] OR “MPDL3280A”[Supplementary Concept] OR “MPDL3280A”[tiab] OR “atezolizumab”[tiab] OR “Tecentriq”[tiab] OR “RG7446”[tiab] OR “RO5541267”[tiab] OR “Durvalumab”[tiab] OR “MEDI4736”[tiab] OR “MEDI-4736”[tiab] OR checkpoint inhibitor*[tiab] OR “PD-1”[tiab] OR “PDL1”[tiab]) AND (Clinical Trial, Phase III[ptyp] OR “phase 3 clinical trial”[tiab] OR “phase III clinical trial”[tiab] OR “phase 3 trial”[tiab] OR “phase III trial”[tiab] OR “phase 3 clinical study”[tiab] OR “phase III clinical study”[tiab] OR “phase 3 study”[tiab] OR “phase III study”[tiab] OR “phase 3 randomized trial”[tiab] OR “phase III randomized trial”[tiab] OR Clinical Trial, Phase II[ptyp] OR “phase 2 clinical trial”[tiab] OR “phase II clinical trial”[tiab] OR “phase 2 trial”[tiab] OR “phase II trial”[tiab] OR “phase 2 clinical study”[tiab] OR “phase II clinical study”[tiab] OR “phase 2 randomized trial”[tiab] OR “phase II randomized  trial”[tiab] OR “phase 2 study”[tiab] OR “phase II study”[tiab] OR “phase 2/3 clinical trial”[tiab] OR “phase II/III clinical trial”[tiab] OR “phase 2/3 trial”[tiab] OR “phase II/III trial”[tiab] OR “phase 2/3 clinical study”[tiab] OR “phase II/ III clinical study”[tiab] OR “phase 2/3 study”[tiab] OR “phase II/III study”[tiab] OR “phase 2/3 randomized trial”[tiab] OR “phase II/III randomized trial”[tiab] OR Randomized  Controlled Trial[ptyp] OR “randomized controlled trial”[tiab]

OR “RCT”[tiab])

**Table S1 Summary of HR for OS by therapeutic regimen and age**

| **Age** | **Anti-PD1/PDL1 monotherapy** | |  | **Anti-PD1/ PDL1 + anti-CTLA4** | |
| --- | --- | --- | --- | --- | --- |
|  | **HR (95% CI)** | **P for interaction *** |  | **HR (95% CI)** | **P for interaction *** |
| < 75 years | 0.69 (0.58 to 0.82) | 0.066 |  | 0.72 (0.62 to 0.79) | 0.329 |
| ≥ 75 years | 0.95 (0.58 to 1.57) |  |  | 0.87 (0.61 to 1.26) |  |

HR, hazard ratio; OS, overall survival; CI, confidence interval;

* P for interaction was expressed as the heteroeneity of efficacy between elderly and non-elderly patients.

**Summary of HR for OS and PFS by age in NSCLC**

| **Age** | **OS** | |  | **PFS** | |
| --- | --- | --- | --- | --- | --- |
|  | **HR (95% CI)** | **P for interaction *** |  | **HR (95% CI)** | **P for interaction *** |
| < 75 years | 0.72 (0.61 to 0.86) | 0.289 |  | 0.77 (0.60 to 1.01) | 0.433 |
| ≥ 75 years | 0.99 (0.67 to 1.30) |  |  | 0.94 (0.67 to 1.30) |  |

HR, hazard ratio; PFS, progression-free survival; OS, overall survival; CI, confidence interval;

* P for interaction was expressed as the heterogeneity of efficacy between elderly and non-elderly patients.


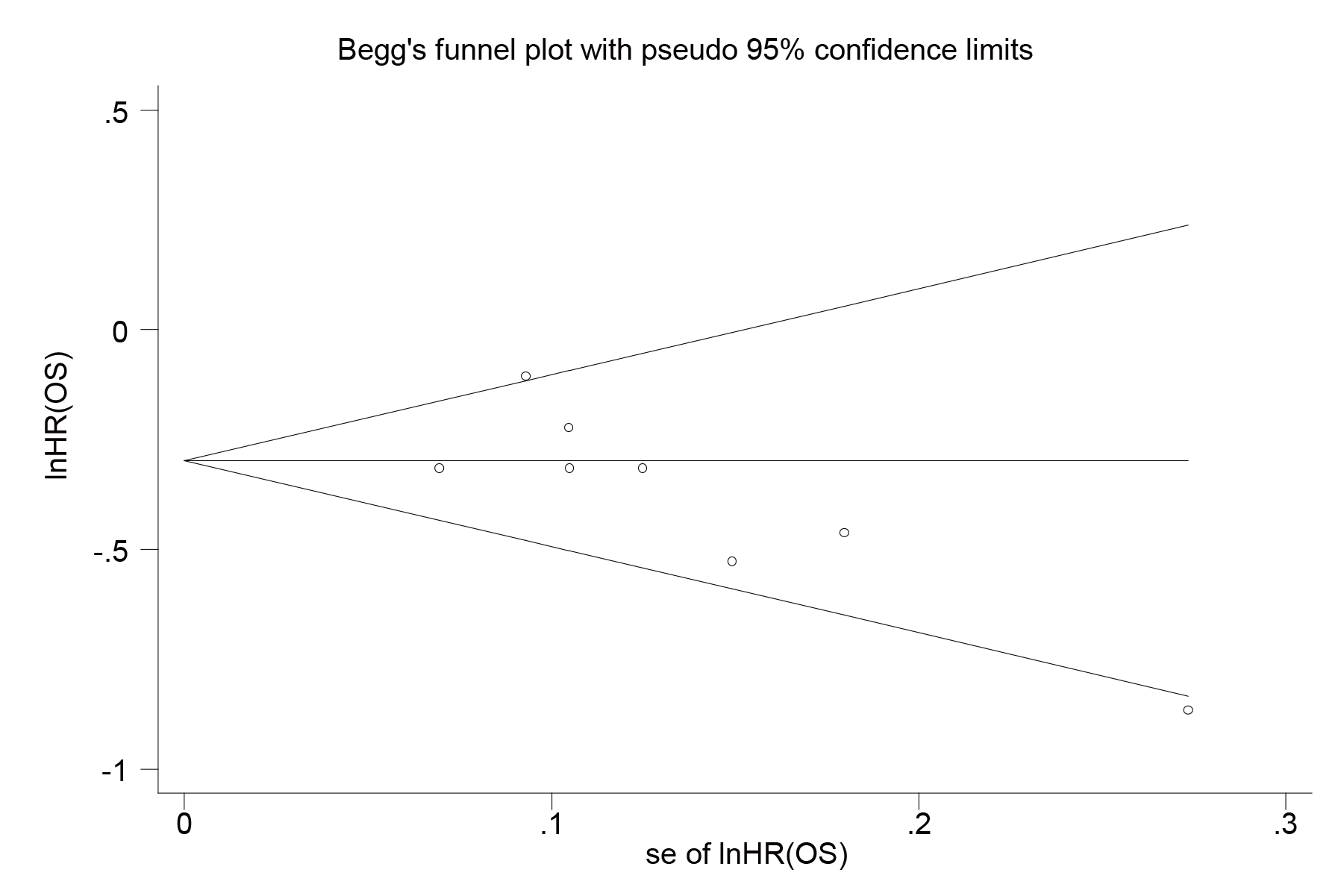


**Figure S1.** Begg’s funnel plot for publication bias test (P = 0.071). Each circle represents a separate study for indicated association, and horizontal line represents the mean effect size.


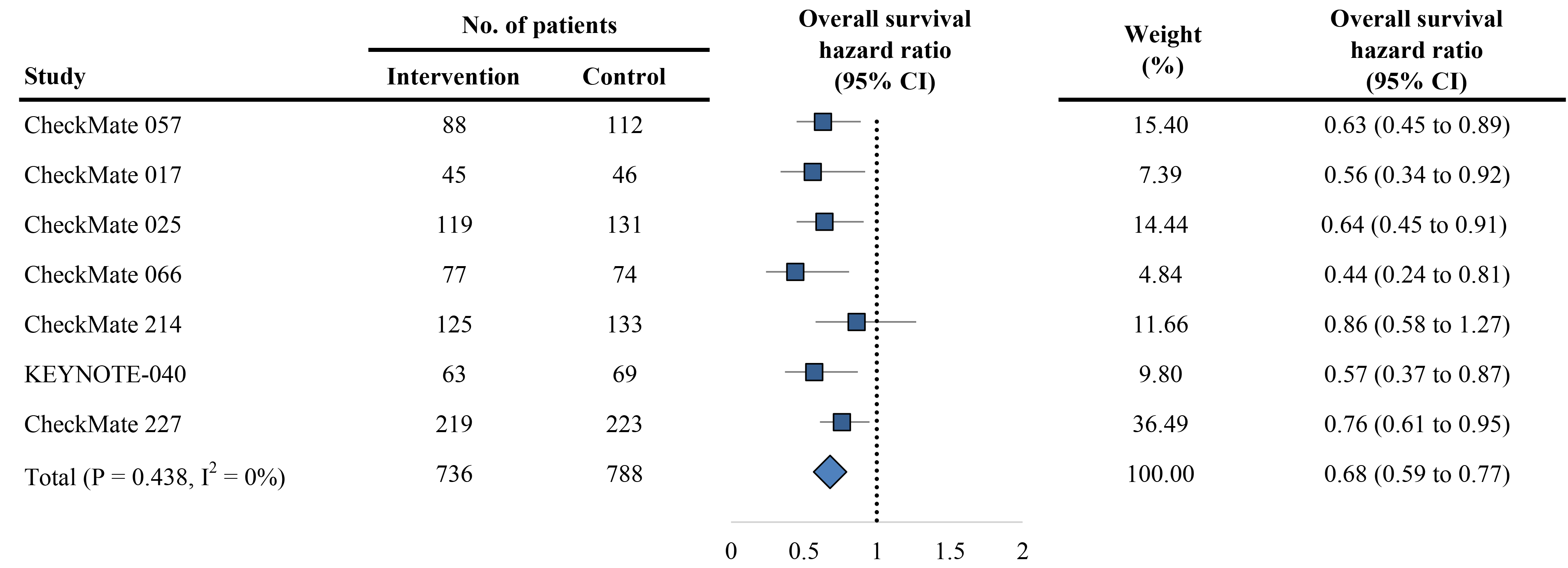


**Figure S2.** Forest plot of hazard ratio for overall survival in patients aged 65-75 years. CI, confidence interval
